# Suppression of Rituximab-resistant B-cell lymphoma with a novel multi-component anti-CD20 mAb nanocluster

**DOI:** 10.18632/oncotarget.4206

**Published:** 2015-06-01

**Authors:** Huafei Li, Ge Zhang, Cheng Jiang, Fulei Zhang, Changhong Ke, He Zhao, Yun Sun, Mengxin Zhao, Di Chen, Xiandi Zhu, Li Zhang, Bohua Li, Jianxin Dai, Wei Li

**Affiliations:** ^1^ International Joint Cancer Institute, the Second Military Medical University, Shanghai, China; ^2^ State Key Laboratory of Antibody Medicine and Targeting Therapy and Shanghai Key Laboratory of Cell Engineering, Shanghai, China; ^3^ PLA General Hospital Cancer Center, PLA Graduate School of Medicine, Beijing, China

**Keywords:** nano mAb's cluster, CD20, apoptosis, non-hodgkin lymphoma, rituximab

## Abstract

Although the anti-CD20 antibody Rituximab has revolutionized the treatment of Non-Hodgkin Lymphoma (NHL), resistance to treatment still existed. Thus, strategies for suppressing Rituximab-resistant NHLs are urgently needed. Here, an anti-CD20 nanocluster (ACNC) is successfully constructed from its type I and type II mAb (Rituximab and 11B8). These distinct anti-CD20 mAbs are mass grafted to a short chain polymer (polyethylenimine). Compared with parental Rituximab and 11B8, the ACNC had a reduced “off-rate”. Importantly, ACNC efficiently inhibited Rituximab-resistant lymphomas in both disseminated and localized human NHL xenograft models. Further results revealed that ACNC is significantly potent in inducing caspase-dependent apoptosis and lysosome-mediated programmed cell death (PCD). This may help explain why ACNC is effective in suppressing rituximab-resistant lymphoma while Rituximab and 11B8 are not. Additionally, ACNC experienced low clearance from peripheral blood and high intratumor accumulation. This improved pharmacokinetics is attributed to the antibody-antigen reaction (active targeting) and enhanced permeability and retention (ERP) effect (passive targeting). This study suggested that ACNC might be a promising therapeutic agent for treatment of rituximab-resistant lymphomas.

## INTRODUCTION

Rituximab (Rtx), the first US FDA (Food and Drug Administration)-approved mAb for treating B-cell non-Hodgkin lymphomas (NHL), targets the CD20 antigen and leads to CD20^+^ B-cell depletion [1–3]. Currently, Rituximab is used in all phases of conventional treatment, including first-line therapy, maintenance, and salvage therapy [4, 5]. Although its clinical effectiveness is uncontested, the effectiveness of Rituximab is ultimately limited partly due to treatment resistance [5, 6]. Only 40% of patients who initially respond to Rituximab will respond again after relapse [7]. Thus, developing novel methods against Rituximab resistance with improved outcome is urgently needed.

Up to now, at least 3 major mechanistic pathways have been proposed by which anti-CD20 mAb causes B-cell depletion, including complement-dependent cytotoxicity (CDC), antibody-dependent cellular cytotoxicity (ADCC) and induction of programmed cell death (PCD) [3]. Anti-CD20 mAbs are often defined as either type I or II mAbs based on their ability to redistribute CD20 into lipid rafts. Most anti-CD20 mAbs described in the literature are Type I mAbs (Rituximab-like), which stabilize CD20 in lipid rafts and induce CDC, but only weakly induce PCD. In contrast, Type II (Tositumomab-like) mAbs do not localize CD20 into lipid rafts and only weakly induce CDC, but appear to induce higher levels of PCD. In addition, both types are comparable in carrying out ADCC [2, 3, 5, 8].

Although all mechanisms mentioned above might be involved in providing therapeutic efficacy, their relative contributions remain unclear [5, 9]. Considering the complex effector mechanisms of Rituximab, the exact mechanisms of Rituximab resistance remain poorly understood. Based on previous publications, the following potential mechanisms might contribute to the resistance: (1) loss of CD20 expression, either through down-regulation or “shaving” of Rituximab/CD20 complexes [10, 11]; (2) exhaustion of effector cells, such as natural killer (NK) cells, etc. [12–14]; (3) host immunologic factors, such as Fc receptor polymorphisms [15–17]; (4) exhaustion of stored complements or increased surface expression of complement control proteins [18–20].

Nowadays, to combat Rituximab resistance, many researches are focusing on the combination therapy of Rituximab with different mAbs. Chao et al's study revealed that anti-CD47 antibodies preferentially enabled phagocytosis of NHL cells and synergized with Rituximab through Fc receptor (FcR)-dependent and -independent stimulation of phagocytosis. Thus the combination therapy led to elimination of lymphoma [21]. Phase I/II studies show that either epratuzumab (a humanized antibody targeting the B cell antigen CD22) or galiximab (a chimeric antibody targeting the costimulatory ligand CD80) in combination with Rituximab demonstrate relative safety. The clinical responses are greater than that of single agent [22–24]. Previously, we constructed an IgG-like bispecific fusion protein targeting both CD20 and Flt3 (CD20-Flex BiFP) and a bispecific mAb targeting both CD20 and HLA-DR (CD20-243 CrossMab) through CrossMab technology, both of which exhibited excellent anti-lymphoma activities [25, 26].

In this study, we successfully constructed an anti-CD20 mAb nanocluster (ACNC) from two different anti-CD20 mAbs, type I mAb Rituximab and type II mAb 11B8. It should be mentioned that nanomedicine is an emerging form of anti-cancer therapy based on the assembling of biological molecules into nano-sized particles [27, 28]. Our experimental results indicate that this nanocluster exhibits strong antitumor activity against Rituximab-resistant B-cell lymphoma. Further results reveal that this nanocluster can induce exceptional potent PCD through both a caspase-dependent and -independent pathway. Moreover, the inter-cell link with ACNC was observed in this study, which was considered to be related with its enhanced PCD evoking ability. Our mAb nanocluster may be a promising strategy for treating Rituximab-resistant B-cell lymphomas.

## RESULTS

### The properties of ACNC nanocluster

**Figure 1:**
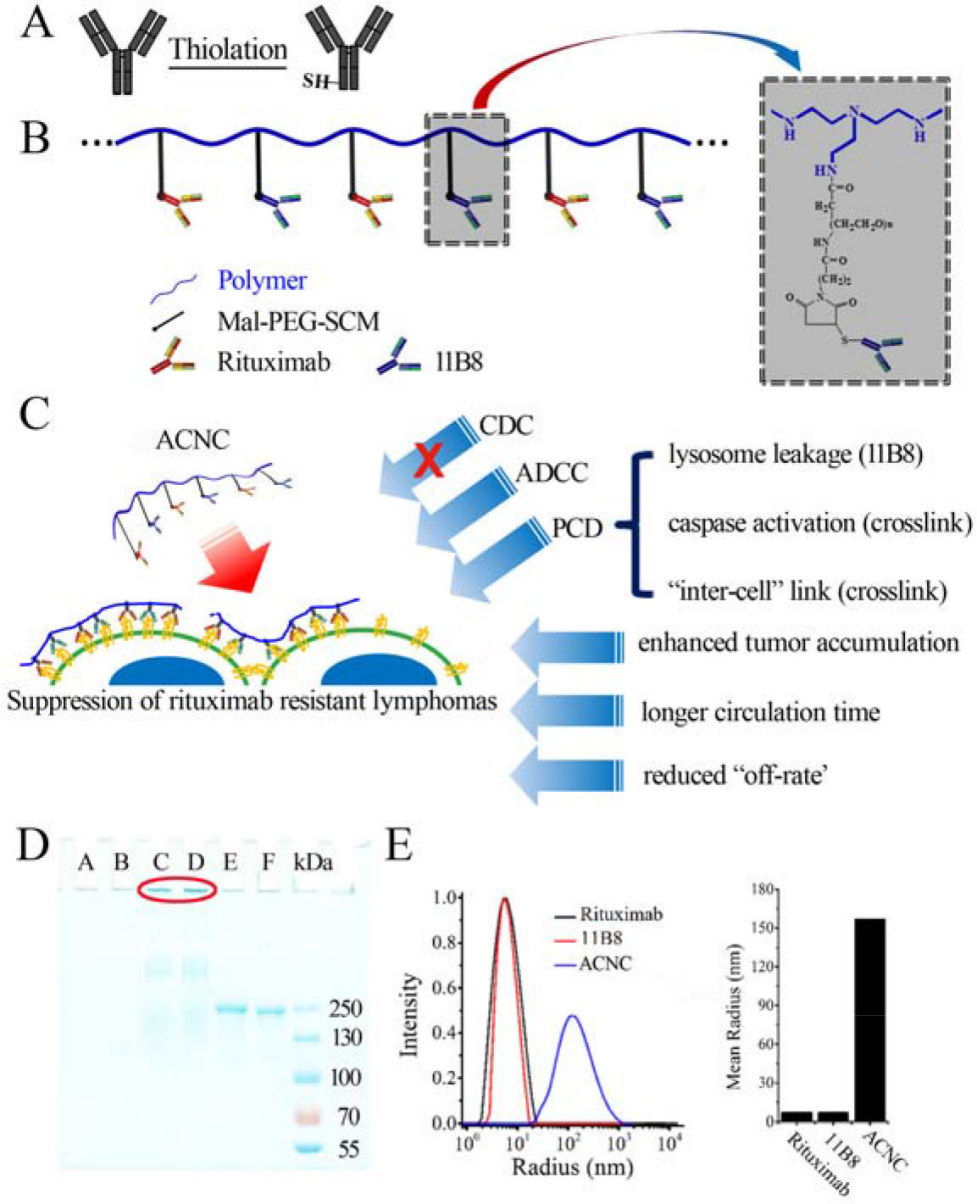
Fabrication and characterization of ACNC **A-B.** Schematic representation of nanoclusters. **C.** Effector mechanisms of ACNC mediated suppression of rituximab-resistant lymphomas. **D.** SDS-PAGE analysis of purified ACNC. Lane A-B: long chain polymer, lane C-D: ACNC; lane E: Rituximab; lane F: 11B8; **E.** Size distribution (left panel) and mean size (right panel) of Rituximab, 11B8 and ACNC.

The properties of ACNC nanocluster were investigated and shown in Figure 1D-1E (which was also confirmed in another system). Its high molecular weight (M_W_) of nanocluster was confirmed by SDS-PAGE (Figure 1D). An obviously stranded protein band of ACNC appeared in lane C-D. On the other hand, no band of polymer in lane A-B and a single band at approximately 150 kDa of unmodified Rituximab and 11B8 respectively in lane E and F. The results were further validated by dynamic light scattering analysis, which showed a mean radius of approximately 176 nm for ACNC, compared with less than 7 nm for Rituximab and 11B8 (Figure 1E).

### Binding avidity of ACNC

**Figure 2:**
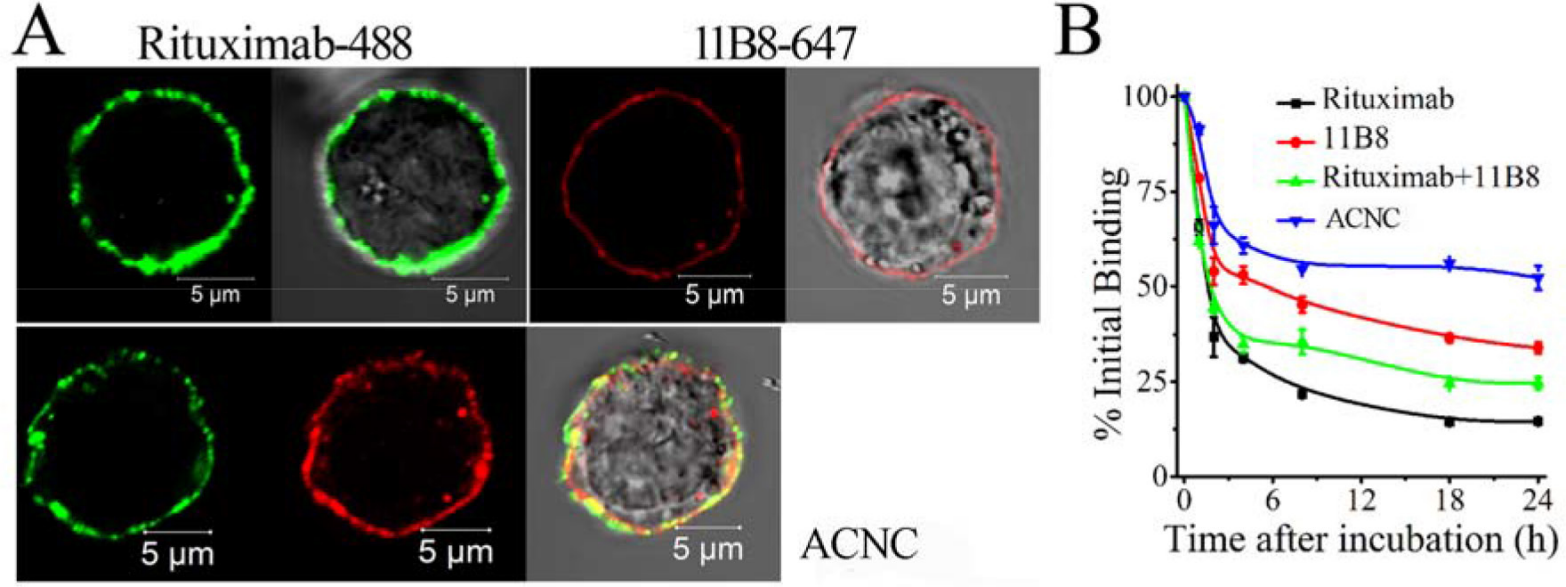
Binding avidity of ACNC to surface CD20 of Raji-anti cells **A.** Raji-anti cells were incubated with 10 μg/ml Alexa Fluor 488/647 labeled Rituximab/11B8 and ACNC on ice for 1 hour and then assessed by confocal microscope. **B.** Dissociation of ACNC and parental mAbs from Raji cells. Cells were incubated with 10 μg/ml Rituximab, 11B8 and ACNC, washed and resuspended in culture medium. Samples were taken at 0, 1, 2, 4, 8, 18 and 24 hours, washed, labeled with GAH-488 and analyzed by FCM. Data are mean ± SD of at least 3 experiments.

Confocal microscopy demonstrated that the exposure of Raji cells to Alexa Fluor-488 labeled Rituximab or Alexa Fluor-647 labeled 11B8 led to decoration of the cell surface with corresponding green or red fluorescence. The exposure to ACNC consisting of both mAbs led to both green and red fluorescence (Figure 2A), which indicated that the bio-recognition between ACNC and CD20 was not affected during the process of fabrication.

The binding “off-rate” experiment was performed to compare the dissociation of ACNC with free mAbs from Raji cells. As shown in Figure 2B, approximately 52.2 ± 3.1% of the ACNC remained on Raji cells after a 24-hour incubation with antibody free culture medium, compared with 14.5 ± 0.1% of Rituximab (**p = 0.001), 33.9 ± 1.4% of 11B8 (**p = 0.002), and 24.6 ± 1.5% of Rituximab + 11B8 (**p = 0.001). These results indicated that 11B8 (type II) owned a reduced “off-rate” compared with Rituximab (Type I) (**p = 0.005). Besides, the ACNC nanocluster showed a much slower “off-rate” than unmodified Rituximab and 11B8 due to the effective crosslink.

### Rituximab-resistant Raji cells failed to respond to Rituximab-induced CDC but not ADCC *in vitro*

**Figure 3:**
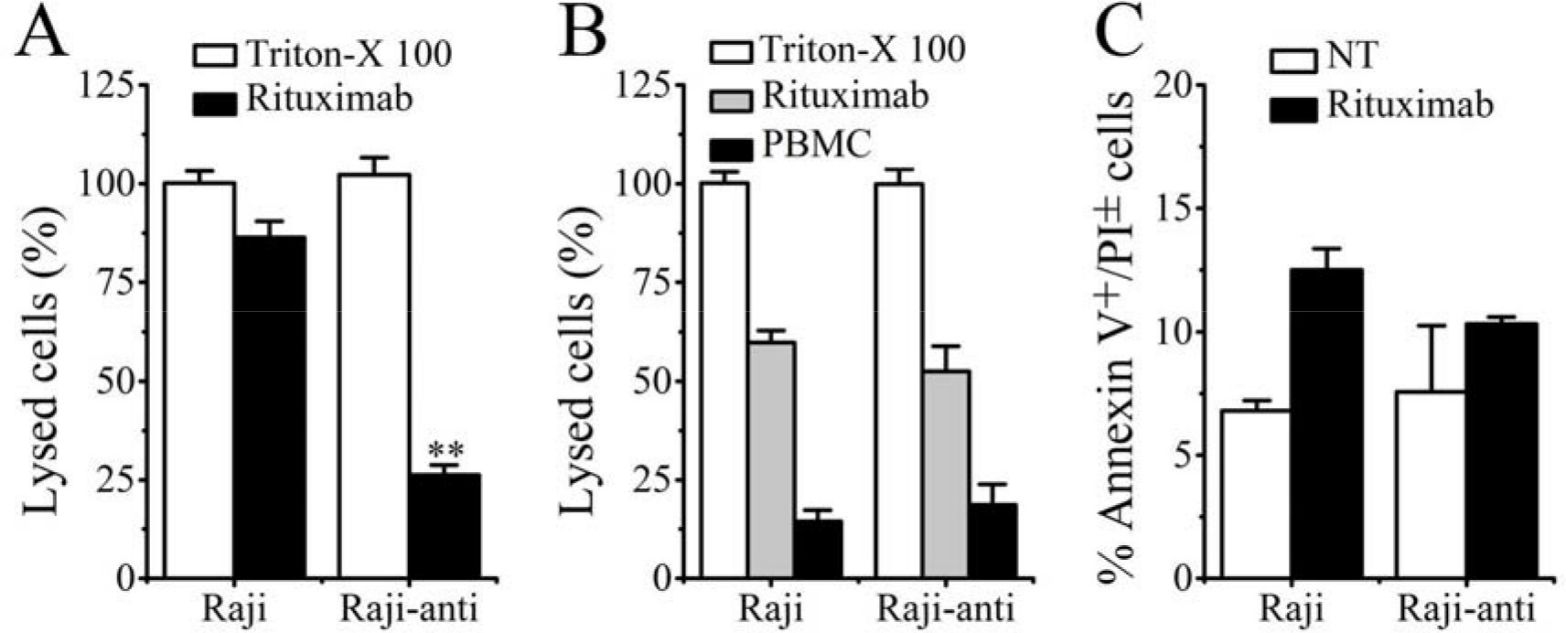
The identification of resistant Raji cells **A.** Rituximab mediated CDC in Raji and Raji-anti cell lines. **B.** Rituximab mediated ADCC in Raji and Raji-anti cell lines. **C.** Rituximab mediated PCD in Raji and Raji-anti cell lines. Data are expressed as means ± SD (*n* = 3), **p < 0.01.

The ability of Rituximab to mediate CDC, ADCC and PCD in resistant clones was subsequently evaluated after the successful generation of Rituximab resistant lymphoma cells (Raji-anti). We further compared it with the WT cells (named as Raji). As shown in Figure 3A, Raji cells exhibited high sensitivity to the cytotoxic effects of Rituximab-mediated CDC. This effect was stepwise augmented by the concentration of Rituximab. Compared with Raji cells, the resistant cells were less sensitive to Rituximab-mediated CDC in all the three tested concentrations (**p = 0.005). However, both of the WT and resistant cells exhibited comparable sensitivity to Rituximab-mediated ADCC (Figure 3B). Besides, Rituximab hardly evoked obvious PCD in WT and resistant Raji clones (Figure 3C).

### ACNC can significantly eliminate resistant lymphomas in both disseminated and localized human NHL Xeno-transplant models

**Figure 4:**
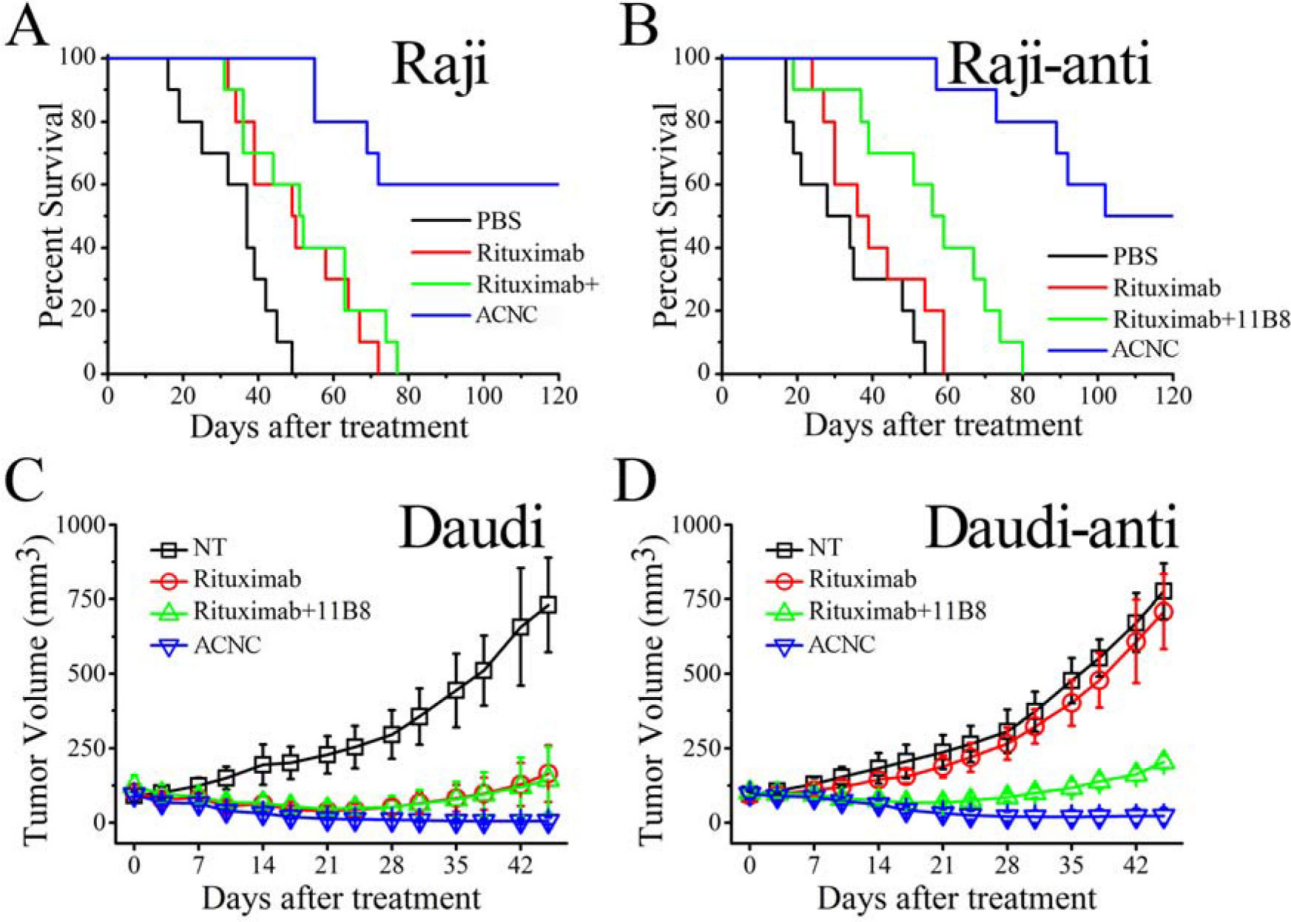
*In vivo* immunotherapy of wild type and rituximab-resistant NHLs by anti-CD20 mAbs and ACNC **A-B.** The survival of ACNC treated SCID mice bearing Raji (A) and Raji-anti (B) cells. **C-D.** Groups of SCID were inoculated subcutaneously with 2 × 10^7^ Daudi (C) and Daudi-anti (D) cells and treated with Rituximab, Rituximab + 11B8 and ACNC. Tumor size was measured 2-dimensionally with a caliper and tumor volume shown as mean ± SD (*n* = 4).

In the disseminated model, Raji and Raji-anti cells were respectively transplanted intravenously into female SCID mice via tail vein. After 5 days, these mice were randomly administered injections of PBS, free Rituximab, Rituximab + 11B8 and ACNC weekly for 3 times. The survival curve is shown in Figure 4A-4B and the results of statistical analysis are shown in Table S1-S2. For the WT Raji cells, the group treated by Rituximab had significantly long survival time than the control group injected by PBS (*p = 0.008). Similar results were seen with combination therapy of Rituximab plus 11B8 (**p = 0.007) and were not statistically different compared to single injection of Rituximab (*p* = 0.494). However, the administration of ACNC can significantly prolong the survival time with a CR percentage of 6/10 indicated by long-term survival (> 120 days post treatment). For the resistant clones, no statistical difference in survival was observed between the treatment of PBS and Rituximab, with a median survival time (MST) of respectively 28 ± 10.28 and 36 ± 7.12 days. Combination therapy of Rituximab and 11B8 can moderately extend the MST to 56 ± 6.33 days (*p = 0.034). However, the mice treated with ACNC had a significantly extended MST of more than 120 days, with statistically significant survival extension by log-rank analysis (**p = 0.01) comparing with the combination therapy of both antibodies. Also, 5/10 mice experienced a complete remission (CR) in ACNC treated group.

The excellent anti-tumor activity of ACNC is validated in a localized model. For the WT lymphomas, Figure 4C revealed that the groups treated by Rituximab ± 11B8 resulted in decreased rate of lymphoma growth. However, the tumor volume of mice treated by ACNC was remarkably suppressed, which was characterized by 3/4 mice of CR having no measurable mass. For the resistant clones (Figure 4D), ACNC treated mice also demonstrated a remarkable decrease in tumor burden measured by tumor volume compared with Rituximab and PBS control treatment, with 1/4 mice showed CR indicated by having no measurable mass. However, immunotherapy by combination of both antibodies can also induce a mild decrease in tumor burden.

### ACNC mediated cell death in resistant lymphoma cells in *in vitro* experiments

**Figure 5:**
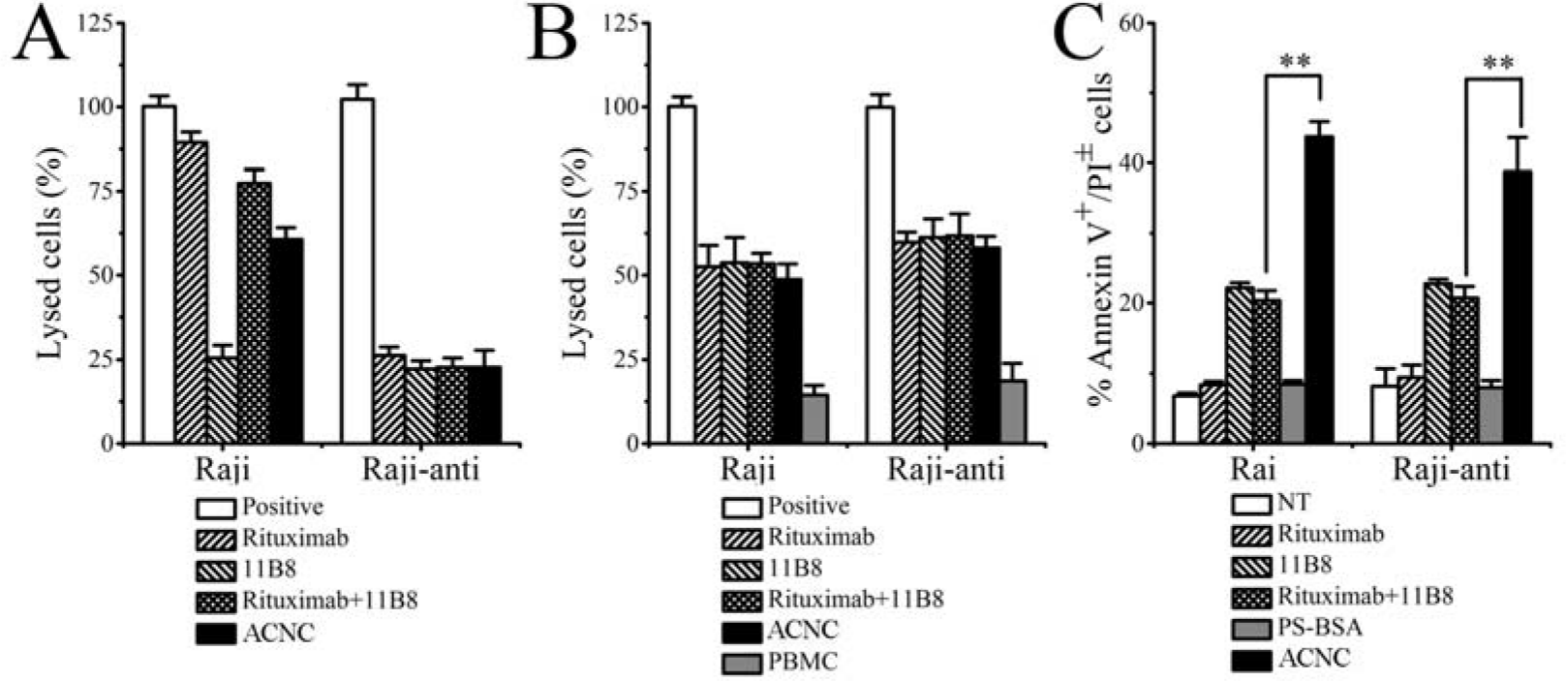
*In vitro* tumor suppression of ACNC against Raji and Raji-anti cells **A.** CDC activity against Raji and Raji-anti cells. Cells were incubated with 10 μg/ml anti-CD20 mAbs and ACNCs, supplemented with 5% (v/v) fresh human serum. Data are expressed as means ± SD (*n* = 3). **B.** ADCC activity against Raji and Raji-anti cells. Cells were incubated with 10 μg/ml anti-CD20 mAbs and ACNCs, supplemented with human PBMCs as effector cells at an E:T ratio of 25:1. Data are expressed as means ± SD (*n* = 3). **C.** PCD-inducing ability of anti-CD20 mAbs and ACNCs. Cells were assessed by FCM following staining with Alexa Fluor 488 anti-Annexin V& PI. Data are expressed as means ± SD (*n* = 3, **p < 0.01).

In order to clarify the exact mechanisms of excellent *in vivo* tumor-inhibitory effect of ACNC on Rituximab-resistant lymphoma, we performed *in vitro* experiments to testify the CDC, ADCC and PCD inducing ability of ACNC. As indicated in Figure 5A, for the high CDC resistance, ACNC and its parental antibodies appeared to be ineffective in inducing CDC in Raji-anti clones. In contrast, their ability to mediate ADCC was not affected (Figure 5B). Figure 5C demonstrated that the Annexin V^+^ subsets induced by free Rituximab and 11B8 was respectively 9.43 ± 1.80% and 22.81 ± 0.65% in Rituximab-resistant Raji cells. However, the nanocluster induced a remarkably higher level of PCD than that induced by combination treatment of parental mAbs (ACNC 38.76 ± 4.88% versus RB 20.72 ± 1.67%, **p = 0.001).

### Involvement of lysosomes in ACNC induced PCD in resistant lymphoma cells

**Figure 6:**
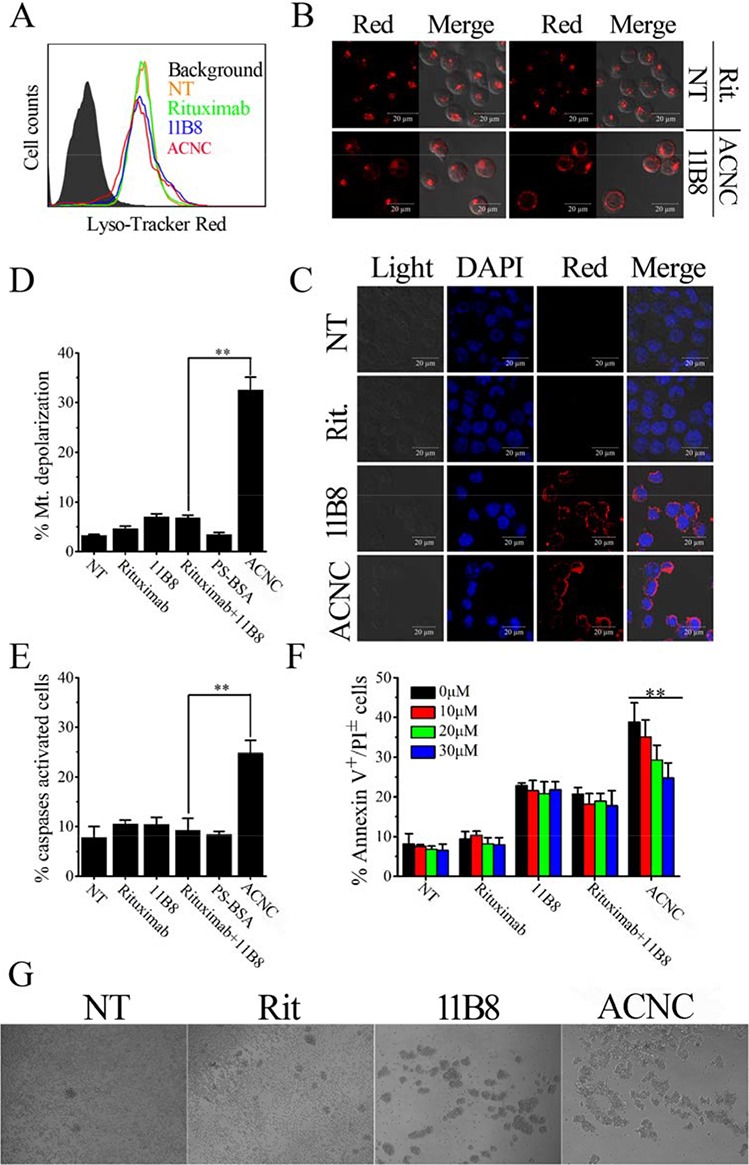
ACNC can induce PCD of Raji-anti cells through both caspase dependent and independent pathways **A-C.** Involvement of lysosomes in ACNC induced cell death. Detection of total lysosomal volume in cells treated with mAbs and ACNCs. The volume of the lysosomal compartment was measured by FCM (A) (Data are obtained in three runs (*n* = 3) (**p < 0.05)) and confocal microscopy (B) after labeling with LysoTracker-red probe. (C) Confocal microscopy of cathepsin B staining (red). DNA was counterstained with DAPI (blue). **D-F.** Involvement of caspases activation in ACNC induced PCD. (D) Detection of mitochondrial membrane potential (MMP) of CD20 mAb and ACNC treated cells. ACNC treated cells were labeled with JC-1 probe and assessed by FCM. Data are expressed as means ± SD (*n* = 3). (E) Caspase activation in ACNC induced PCD. Cells were stained with a FLICA reagent provided and assessed by FCM. The percentage of caspases activated cells among groups was calculated and shown as mean ± SD (*n* = 3). (F) ACNC induced PCD can be partly but not completely inhibited by a caspases inhibitor (Z-VAD-FMK). Prior to the addition of mAbs and ACNCs, Cells were treated for 30 minutes with 0, 10, 20, 30 μM Z-VAD-FMK. Apoptotic cells were assessed as previous description. Data are expressed as mean ± SD (*n* = 3) (**p < 0.01). **G.** Identification of “inter-cell” crosslink of ACNC. Cells were treated with 2.5 μg/ml Rituximab, 11B8 and ACNC for 8 hours. Then cell morphology was observed by light microscopy.

Previous studies revealed that lysosome plays an important role in type II anti-CD20 mAb induced PCD [37]. In order to characterize the involvement of lysosomes in ACNC induced PCD, lysosome tracker was used to label the targeting resistant cells (Figure 6A). FCM results revealed that the distribution of FL-2 (lyso-tracker) in Raji-anti cells experienced a visible red shift, including increase and decrease of red fluorescence intensity, after a 16-hour-incubation with 11B8 (blue histograms) and ACNC (red histograms) compared to NT group (orange histograms) or Rituximab treatment (green histograms). CLSM images results (Figure 6B) clearly explained this discrepancy, which revealed that in normal cells, cellular lysosomes were labeled as relatively small and confined organelles, while after 11B8 or ACNC treatment, an enlargement of red fluorescence labeled compartments and diffusion of red fluorescence in the cytoplasm was detected. Considering previous publications [37, 38], we believed these cells were successively undergoing a swelling of lysosomes (enlargement of red fluorescence labeled compartments) and the collapse of lysosomal compartment (diffusion of red fluorescence in the cytoplasm).

For further validation of lysosome compartment collapse, we performed IF staining for cathepsin B, which is known as a lysosomal component (Figure 6C). Confocal microscope revealed that a substantial increase in cathepsin B (red fluorescence) was found throughout the cytoplasm of ACNC treated cells, in accordance with the results of lysosome tracker labeling.

### Involvement of caspase activation in ACNC induced PCD

For the determination of caspase involvement in ACNC induced PCD, the mitochondrial depolarization was firstly evaluated by FCM post JC-1 staining, cells undergone mitochondrial depolarization was indicated by decreased fluorescence intensity in FL-2 (JC-1 red). As shown in Figure 6D, neither free Rituximab nor 11B8 can cause significant changes in mitochondrial membrane potential (MMP), while ACNC treated cells experienced a remarkable mitochondrial depolarization in resistant Raji cells.

In accordance, Figure 6E revealed that the caspase can hardly be activated by free anti-CD20 mAbs in Raji-anti cells. However, a significant increase of caspase activation appeared as a result of ACNC treatment compared with the combination treatment of free parental antibodies (**p = 0.002). PCD inhibition results (Figure 6F) revealed that ZVAD-FMK (a caspases inhibitor) over a range of concentrations from 10 to 30 μM can hardly prevent free antibody-induced PCD. While ACNC evoked PCD can be significantly but not completely reduced by ZVAD-FMK (**p = 0.001). These results clearly demonstrate that ACNC can successfully induce PCD via both a caspase dependent and independent manner in resistant lymphoma cells.

### Inter-cellular clusters may play an important role in ACNC induced PCD in resistant lymphoma cells

Our previous observations (the work the mass arrayed antibodies can evoke that will be submitted soon) have found large inter-cellular clusters than free antibodies. In order to confirm this interesting phenomenon, Raji-anti cells were incubated with 2.5 μg/ml anti-CD20 mAbs or ACNCs. As displRituximab post observed were small cell-clusters ayed in Figure 6G, few treatment compared with some larger ones post 11B8 treatment. Surprisingly, the largest inter-cellular clusters appeared among cells as treated by the mAb nanocluster. This obvious inter-cellular cell clusters was similar to Homotypic Adhesion (HA) [36], which we thought can contribute to ACNC induced PCD as discussed in the following section.

### Pharmacokinetics assays

For pharmacokinetics (PK) assays, one-compartment model was used to describe the time course of blood concentrations. The PK parameters was demonstrated in Table 1, which revealed that the elimination of ACNC from mouse peripheral blood is much slower than that of free antibodies, with a slower clearance (CL) (ACNC: 7.49 ± 1.01L/h versus Rituximab 12.13 ± 2.04L/h and 11B8 11.83 ± 0.84L/h, **p = 0.001) and longer elimination half-time (t_1/2_) (ACNC: 429.0 ± 37.6H versus Rituximab 274.8 ± 30.1 h and 11B8 278.4 ± 9.0 h, **p = 0.001).

**Table 1:** Parameter of pharmacokinetics

Parameter	Rituximab	11B8	ACNC
**t_1/2_ (h)**	274.8 ± 30.1	278.4 ± 9.0	395.0 ± 51.6
**CL(L/h)**	12.13 ± 2.04	11.83 ± 0.84	8.47 ± 1.47
**MRT (h)**	396.52 ± 43.41	401.58 ± 13.02	569.91 ± 74.48
**V_d_ (ml)**	4.75 ± 0.27	4.74 ± 0.21	4.75 ± 0.23

t_1/2_: Elimination Half-life

CL: Clearance

MRT: Mean residence time

Vd: Apparent volume of distribution

### *In vivo* distribution

**Figure 7:**
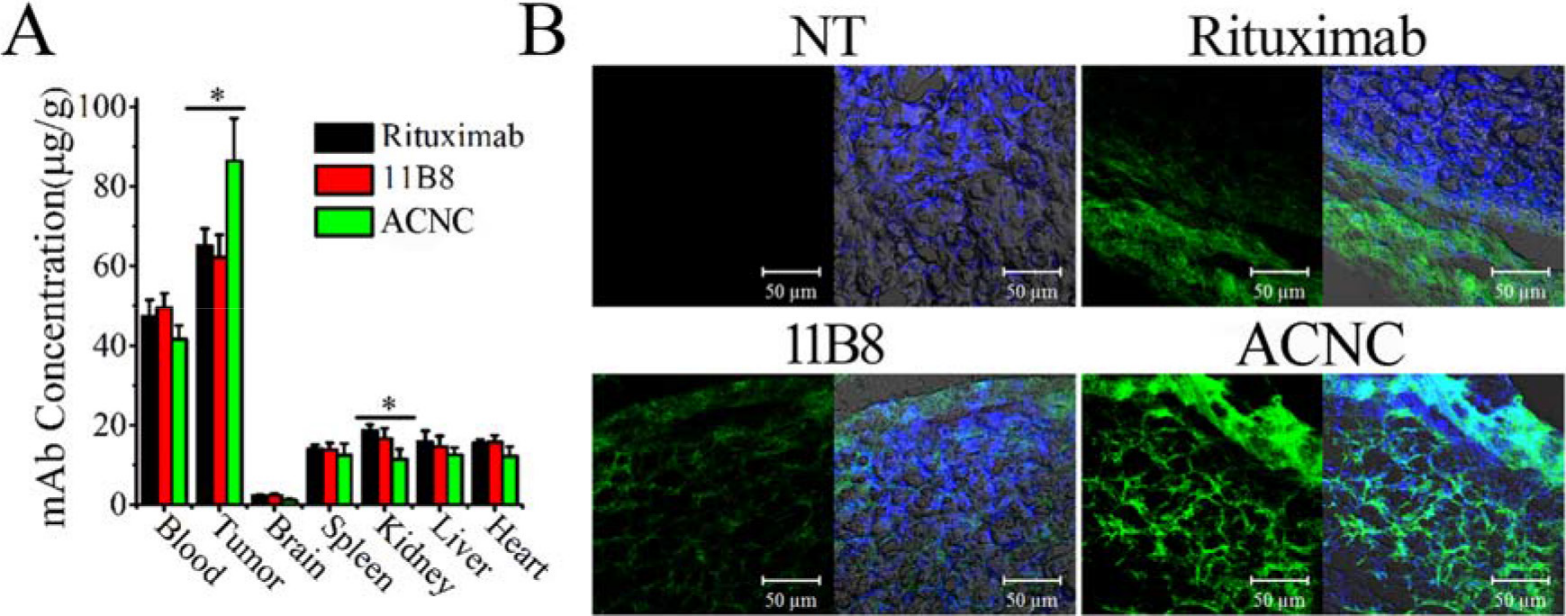
*In vivo* distribution ACNC in lymphoma bearing SCID mice **A.** Therapeutic mAb concentration in different tissue samples were determined by ELISA. Data are the mean ± SEM derived from separate organs of three different animals. **B.** Frozen section from tumors were stained by DAPI (blue) and Alexa Fluor 488 goat anti-human secondary antibodies (green) as visualized and assessed by confocal microscope.

Lymphoma (Daudi-anti) bearing mice were administrated tail vein injection of ACNC and free mAbs. After 24 hours, mice were sacrificed and the major organs, including tumor, brain, blood, heart, kidney, liver and spleen, were assayed for mAb concentration, which is expressed as μg/g (mAb/tissue) and graphically presented in Figure 7A. Significant increases in the accumulation of ACNC were observed compared with free Rituximab (*p = 0.023) and 11B8 (*p = 0.013). Moreover, the kidney concentration of ACNC experienced a significantly decrease compared with free Rituximab (*p = 0.014) and 11B8 (*p = 0.045). The drug concentration of ACNC seems to be lower than free antibodies in all the other organs, although no statistical difference was found. Additionally, the frozen sections of tumor tissues were prepared for IF staining and observed by CLSM (Figure 7B). In keeping with the ELISA results, the tumor accumulation of ACNC was significantly increased with much higher green color.

## DISCUSSION

Although the clinical effectiveness of Rituximab is uncontested, a significant number of patients gradually become unresponsive after repeat treatment. The phenomenon was termed as rituximab resistance, which is one of the biggest challenges to solve in the clinic [5, 6]. As we all know, cancer is usually multifactorial in nature, involving various redundant disease-mediating ligands and receptors, as well as crosstalk between signal cascades [39, 40]. Thus, targeting only one or two pathways may not completely shut off a hallmark capability of cancer. This allows some malignant cells to survive until they or their progeny eventually adapt to the selective pressure [25, 26]. Therefore, the activation of multiple tumor suppression pathways may result in improved therapeutic efficacy and reduce drug resistance. Moreover, the combination of different antibodies should be better than single antibody.

In this study, we successfully constructed an anti-CD20 antibody nanocluster (ACNC) from Rituximab (type I) and 11B8 (type II). The details of the construction were shown in Figure 1. Shortly, this cluster formed by ACNC can bind to surface CD20 of lymphoma cells with affinity similar to that of parental antibodies. It is known that both the PEI and antibody are hydrophilic macromolecules. It is known that hydrophilic macromolecule will be in a coil state in aqueous solution. A hydration layer will thus form and surround the polymer coil, which contribute to its stability [41]. For the ACNC nanocluster, its stability is also dominated by this hydration layer in the physiological environments. On the other hand, its binding “off-rate” significantly reduced because of efficient cross-linking by PEI polymer, which may contribute to its long-lasting anti-tumor activity. The “off-rate” reflects the binding ability of the antibodies. For the ACNC, many mABs were arrayed along one single polymer chain. The linking of the mAbs with each other increased the size of ACNC. The larger size will lead to the higher possibility of mAb binding with the antigens on the cellular surface. This consequently hampered the neighboring mAb disassociation from the cellular surface, which thus reduced the disassociate constant of antibody resulting in low off-rate.

In order to determine the anti-tumor efficacy against Rituximab resistance lymphomas, Rituximab resistant cell lines were generated from wild type cells by exposing to fresh human serum and stepwise increased concentrations of Rituximab. The experimental results revealed that the Raji-anti clones were unresponsive to Rituximab mediated CDC but not ADCC. As evaluated by the Rituximab sensitive Raji cells (Raji), we can find that the percentage of lysed cells treated by Rituxmab was about 85%. This value decreased to ~ 60% when Raji cells were treated by ACNC at the same conditions. Such CDC was almost not affected by this chemical crosslink in the right part in Figure 5 A because the data was obtained in the Rituximab resistant Raji cells (Raji-anti). However, the modification of Fc fragments had little effects on the ADCC function, which was shown in Figure 5B. The similar results were also reported in others’ work [42]. Previous studies suggested that the up-regulation of complement regulation proteins (CRPs) such as CD55 and CD59 on surface of lymphoma cells inhibits the activation of complement systems, which might be the main cause of resistant cells’ unresponsiveness to Rituximab mediated CDC *in vitro* [43, 44]. However, the mechanism of Rituximab resistance *in vivo* is much more complex. The exhaustion and disability of complement and effector cells as well as host immunologic factors (such as Fc receptor polymorphisms) may limit the efficacy of Fc related ADCC and CDC evoked by Rituximab [12–20]. The PCD inducing ability was not independent of Fc fragments and limited complement and effector cells. So enhancing PCD inducing ability might be an effective strategy for suppressing Rituximab-resistant lymphomas.

*In vivo* studies illustrated that ACNC was more effective than free mAbs in curing both wild type and rituximab-resistant B cell lymphomas in both disseminated and localized human NHL Xeno-transplant models. Subsequently, we carried out experiments to clarify the tumor suppressing mechanisms against resistant NHLs. As shown in Figure 5, our experimental results indicated that although resistant Raji cells are unresponsive to anti-CD20 mAb mediated CDC, ACNC can mediate exceptional potent PCD in rituximab resistant lymphoma cells *in vitro.* As for the enhanced PCD inducing ability, the lysosome leakage mediated by 11B8 (type II) undoubtedly plays an important role. But even more important is the caspase activation mediated by the efficient crossilink of CD20 antibody-antigen complex, which can only be realized by a secondary antibody in *ex vivo* but not *in vivo* experiments previously [2, 3, 45]. Interestingly, we accidentally found that comparing with free antibodies, lager cell clusters was observed in ACNC treated cells. Because a great many of antibodies tightly anchored to one nanocluster, we consider that ACNC owns the ability of binding to different CD20s on distinct cells. This phenomenon we termed as “inter-cell link”. Such “inter-cell link” may be the nature of the lager cell clusters, which was similar to the HA in previous publications [37]. Many studies suggested that the increased HA of malignant cells might result in increased susceptibility to PCD [46, 47]. Moreover, mAb evoked HA of lymphoma cells can trigger intracellular changes including mitochondrial depolarization, lysosomal membrane permeabilization and phosphorylation or up-/down-regulation of proteins related to the PCD signal transduction pathways, culminating in cell death [26, 37, 38, 48]. Noted here, the mechanism about resistance suppression by the ACNC may be related to many reasons like the CDC, PCD or direct cell death. Herein, the mechanism related to ACNC's function mainly due to the caspase-dependent and -independent programmed cell death (PCD), or termed as direct cell death (DCD).

Further pharmacokinetics results revealed that the elimination of ACNC from mouse peripheral blood is much slower than that of free antibodies. The slower clearance (CL) and longer elimination half-time (t_1/2_) can result in longer circulation time of ACNC in the blood vessels, which can contribute to its durable tumor suppressing abilities. The *in vivo* distribution analysis revealed a significant increase in the accumulation of ACNC compared with free Rituximab and 11B8. The obviously promoted tumor accumulation and retention is attributed to the antibody-antigen identification and combination (active targeting) [49] and the enhanced permeability and retention effect (EPR) (passive targeting) [50]. Additionally, both the longer circulation time (Table 1) and the reduced “off-rate” (Figure 2) may also enhance intratumor accumulation (Figure 7). Noted here, the EPR effect mainly refers to property that some certain sizes of molecules such as liposomes, nanoparticles, and macromolecular drugs tend to accumulate in tumor tissue much more than they do in normal tissues. But the traditional EPR effect concept may be convenient misconceptions as discussed in the excellent perspective [51]. And further analyzed the data discussed in the reference [52], it was found that only 5% of particle (drug conjugate) accumulated in tumor and about 90% accumulated in liver and spleen. But about 7% of albumin and IgG accumulated in tumor and less than 10% accumulated in liver and spleen. So they claimed that the tumor accumulation of IgG or albumin may be better than others. In this study, the ACNC is mainly constructed by the antibodies. Thus, the enhanced tumor accumulation may mainly attribute to the antibody-antigen identification and combination and multivalent binding, with part of the convenient EPR. The detail mechanism was also shown in Figure 1C.

In conclusion, we connstructed novel anti-CD20 mAb's nanoclusters by a facile strategy, which contained anti-CD20 mAb nanocluster (ACNC) from two different anti-CD20 mAbs, type I mAb Rituximab and type II mAb 11B8. Our experimental results indicate that this nanocluster, which is extremely potent in PCD, exhibits strong antitumor activity against Rituximab-resistant B-cell lymphoma. Systemic *in vitro*/vivo investigation revealed that this nanocluster can induce exceptional potent PCD through both a caspase-dependent and -independent pathway. Moreover, the inter-cell link with ACNC was observed in this study, which was considered to be related with its enhanced PCD evoking ability. The results clearly demonstrate that this anti-CD20 mAb nanocluster shows a unique ability to inhibit Rituximab-resistance lymphomas. Our mAb nanocluster may be a promising strategy for treating Rituximab-resistant B-cell lymphomas, which suggested that it warrants further evaluation as cancer therapeutics in the clinic.

## MATERIALS AND METHODS

### Cell lines, materials and animals

Human B-lymphoma cell lines, Raji and Daudi, were obtained from the American Type Culture Collection. Cells were routinely propagated and maintained in RPMI 1640 supplemented with 10% (v/v) heat-inactivated fetal bovine serum (FBS, GIBCO, USA). Rituximab was purchased from Roche and 11B8 was expressed and purified in our laboratory as described previously [29]. The rabbit anti-human IgG F(ab’)2 fragments (RAH) and Polyethylenimine (PEI) polymer were purchased from Sigma-Aldrich (USA). Four-week-old healthy female SCID mice and BALB/C nude mice were purchased from Shanghai Experimental Animal Center of Chinese Academic of Sciences (Shanghai China), housed in specific pathogen-free conditions (SPF) and treated in accordance with guidelines of the Committee on Animals of the Second Military Medical University (Shanghai China).

### Fabrication and characterization of anti-CD20 mAb nanoclusters

The detail preparation process and properties of anti-CD20 mAb nanoclusters was schemed in Figure 1A–1C. Short chain PEI polymer (25kDa, PS) was dissolved in phosphate buffered saline (PBS) and added to maleimide-PEG-SCM (MPEGS, Greative PEGworks, USA) solutions with a PEI/MPEGS ratio of 1:20. After stirring for 4 hours at room temperature (RT), unreacted MPEGS was separated by dialysis. The Rituximab ~ SH, 11B8 ~ SH and BSA ~ SH were produced as described in previous publications [30]. Then equal amount of thiolated Rituximab and 11B8 were mixed and dropped into a 5 ml test tube containing the MPEGS-PEI solution (mAb/PEI mass ratio = 1000:3.44). The reaction was carried out in N_2_ environment for 6–8 hours and uncombined mAbs were separated by dialysis. The PEI-MPEGS-BSA (PMP-BSA) solutions were constructed in the same way. Purified ACNC was quantifed by Nano VueTM (GE Healthcare) and analyzed on 8% SDS-PAGE followed by Coomassie Brilliant Blue staining [30].

### Generation of Rituximab resistant Raji cells

The Rituximab-resistant Raji and Daudi cells were generated as described previously [31] with some minor revisions. Briefly, wild type (WT) cells were exposed to Rituximab at a starting concentration of 0.125 μg/ml in RPMI-1640 containing 5% fresh human serum overnight and then grown in normal culture medium for another 4–5 days for recovery. The concentration of Rituximab was increased by two times every week for 11 cycles until 128 μg/ml. The cells were named by their parental cells with the final concentration of Rituximab to which they were resistant (such as Raji-anti/1.125 μg/ml). For the sake of simplicity, the cells resistant to 128 μg/ml Rituximab was just named as Raji-anti or Daudi-anti for short.

### Confocal microscopy

For confocal microscopy, harvested cells were placed onto poly-D-lysine (sigma-Aldrich) coated microscope slides, fixed in 4% paraformaldehyde, and permeabilized by 0.3% Triton X-100. After staining, samples were observed using a confocal microscope (Zeiss, Germany). Noted here, the artefact appeared in FC and confocal, which may be induced by the antibodies or some HA as mentioned by Golay et al [32]. So in the experiments of confocal or FC, the samples were carefully purification and checked by the inversed fluorescent microscopy to ensure no artefacts existed in the system.

### Binding avidity assays

The binding avidity was assessed by confocal microscopy. Briefly, 10 μg/ml Rituximab, 11B8 or ACNC was incubated with harvested cells for 1 hour on ice, rinsed with PBS and labeled with Alexa Fluor 488 goat anti-human IgG secondary antibody (GAH-488, 1:500, Invitrogen). After washing, the stained cells were observed using a confocal microscope (Zeiss, Germany).

### Off-rate measurement

Harvested cells were incubated with 10 μg/ml Rituximab, 11B8 or ACNC for 1 hour. Then cells were washed and resuspended in mAb free medium. After different time intervals, samples were collected, stained with GAH-488 and analyzed by FCM. The percentage of initial binding was calculated by the following equation:

$$\text{Initial Binding }\%=\left(\frac{{\text{MFI}}_{\text{sample}}-{\text{MFI}}_{\text{NT}}}{{\text{MFI}}_{0h}-{\text{MFI}}_{\text{NT}}}\right)\;\times \;100\%$$

### Immunotherapy

For the establishment of Raji or Raji-anti tumor mouse model, four groups of 10 eight-week-old female SCID mice were injected via the tail vein with 1 × 10^7^ Raji-WT or Raji-anti cells. After 72 hours, mice were randomly administered through tail vein by injections of Rituximab, Rituximab + 11B8 (1:1) and ACNC (15 mg/kg) every other day for 5 times. The mice were observed daily until natural death in a range of 120 days. All animals survived were sacrificed at 120 days.

For the establishment of Daudi or Daudi-anti tumor mouse model, Daudi-WT or Daudi-anti cells (2 × 10^7^) were noculated subcutaneously into the lateral flank of 8-week-old female SCID mice. When the tumors reached about 8 mm in length, 20 mg/kg Rituximab, Rituximab + 11B8 (1:1) or ACNC was intravenously injected though tail vein weekly for 3 times. The tumor size was measured in two perpendicular diameters with precision calipers twice a week and calculated by the following equation:

$$\text{Tumor Volume}=\text{Length}*\;{\text{Width}}^{2}/2$$

### Annexin V & PI staining

Rituximab, 11B8 and ACNC treated cells were stained with Alexa Fluor-488 Annexin-V&Propidium Iodide (PI) (Invitrogen) and analyzed by two channel-FCM of FL-1 (Annexin-V) and FL-2 (PI) following the product information. For PCD inhibition assays, a caspases inhibitor (Z-VAD-FMK, Promega) with different concentrations was added before the addition of mAbs or ACNCs.

### CDC and ADCC assays

Cells were incubated with different concentrations of anti-CD20 mAbs or ACNCs. For CDC assays, 5% (v/v) fresh human serum was added as a source of complement. For ADCC assays, human peripheral blood mononuclear cells (PBMCs) were added as effector cells (effector/targe*t* = 25:1). After a 4-hour-incubation, the lysed cells were measured by the mean luminescence intensity (MLI) determined by Synergy2 Multi-Mode Microplate Reader (Bio Tec, Vermont USA) using CytoTox-Glo TM Cytotoxicity Assay kit (Promega) following the product information.

### Lysosomal permeability assessment

Cells were labeled with 200 nM Lyso-Tracker Red DND (Invitrogen) at 37°C for 30 minutes after treating with 10 μg/ml anti-CD20 mAbs or ACNCs for 16 hours. Then FL-2 fluorescence of labeled cells was assessed by FCM and confocal microscope. Unlabeled cells were used as a background control [33].

### Mitochondrial membrane potentials (MMP) and caspases activation assays

Cells were incubated with anti-CD20 mAbs and ACNCs for 16 hours. After washing, JC-1 probe (Beyotime Biotechnology, Shanghai China) and the Vybrant^®^ FAM Poly Caspases Assay Kit (Invitrogen) were respectively employed to measure mitochondrial depolarization and caspases activation of Raji cells by FCM following the product information.

### Homotypic Adhesion (HA) measurement

Cells were incubated with 2.5 μg/ml anti-CD20 mAbs or ACNCs for up to 8 hours and cell morphology was observed by light microscopy. For confocal microscopy, cells were carefully transferred onto poly-D-lysine coated microscope slides, labeled with 1:500 GAH-488 and assessed by a confocal microscope.

### Pharmacokinetic analysis

Three groups of 3 BALB/C nude mice were injected via the tail vein with 20 mg/kg Rituximab, 11B8 and ACNC respectively on day 0, 1 and 2. After different time intervals, 40–60 μl venous blood was taken from the angular vein of the eyes. Enzyme-linked immunoassays (ELISA) were employed to investigate the plasma concentration of therapeutic mAbs following previous studies [34, 35]. The data were analyzed by the PK solver software [36].

### *In vivo* distribution analysis

Daudi-anti cells (2 × 10^7^) were inoculated subcutaneously into the lateral flank of 8-week-old female SCID mice. When the tumors reached approximately 8 mm in length, 20mg/kg Rituximab, 11B8 or ACNC were intravenously injected though tail vein daily for 3 times. After 24 hours, mice were sacrificed. The organs were collected and the frozen sections stained by GAH-488 were visualized by the CLSM. Meanwhile, different tissues were added with cell lysis buffer and lysed by the tissuelyser-24 (Shanghai jingxin experimental technology, Shanghai China). After centrifugation, ELISA was employed to investigate the supernatant concentration of therapeutic mAbs.

### Statistical analysis

Statistical analysis was performed by Student's unpaired *t* test or ANOVA to identify significant differences unless otherwise indicated. Differences were considered significant at a *p* value of less than 0.05. ANOVA was used for comparing the difference among three or more groups in the experiments.

## SUPPLEMENTARY TABLES


