# Supplementary material for: Suppression of Rituximab-resistant B-cell lymphoma with a novel multi-component anti-CD20 mAb nanocluster
Source: Oncotarget. 2015 Jun 1;6(27):24192–204. doi: 10.18632/oncotarget.4206 (PMC4695179; doi:10.18632/oncotarget.4206)
Supplement: Supplementary file 1 [file oncotarget-06-24192-s001.pdf]

SUPPLEMENTARY TABLES

Supplementary Table S1. Survival analysis by log rank test (Raji-WT cells)

	Pair Comparisons of survival						Median survival time		
Group	Rituximab		Rituximab + 11B8		ACNC				
	Chi-Square	*p* value	Chi-Square	*p* value.	Chi-Square	*p* value	Mean	SD	95% CI
PBS	7.031	0.008	7.314	0.007	21.769	0.000	37.00	3.87	29.41 ~ 44.59
Rituximab	——	——	0.469	0.494	12.154	0.000	49.00	8.70	31.95 ~ 66.05
Rituximab + 11B8	——	——	——	——	10.177	0.001	51.00	6.33	38.60 ~ 63.40
ACNC	——	——	——	——	——	——	> 120	——	> 120~

CI: Confidence Interval
p: significance

Supplementary Table S2. Survival analysis by log rank test (Raji-R cells)

Pair Comparisons of survival							Median survival time (Day)		
Group	Rituximab		Rituximab + 11B8		ACNC		Mean	SD	95% CI
	Chi-Square	*p*	Chi-Square	*p*	Chi-Square	*p*			
PBS	1.910	0.167	9.359	0.002	21.940	0.000	28.00	10.28	7.86 ~ 48.14
Rituximab	——	——	4.506	0.034	19.187	0.000	36.00	7.12	22.05 ~ 49.95
Rituximab + 11B8	——	——	——	——	15.117	0.000	56.00	6.33	43.60 ~ 68.39
ACNC	——	——	——	——	——	——	> 120	——	> 120 ~

CI: Confidence Interval
p: significance
